# Genetic Dissection of Salt Tolerance and Yield Traits of *Geng* (*japonica*) Rice by Selective Subspecific Introgression

**DOI:** 10.3390/cimb45060305

**Published:** 2023-05-31

**Authors:** Simin Li, Ting Feng, Chenyang Zhang, Fanlin Zhang, Hua Li, Yanjun Chen, Lunping Liang, Chaopu Zhang, Wei Zeng, Erbao Liu, Yingyao Shi, Min Li, Lijun Meng

**Affiliations:** 1 College of Agronomy, Anhui Agricultural University, Hefei 230036, China; lisimin@stu.ahau.edu.cn (S.L.); 20720156@stu.ahau.edu.cn (T.F.); zhangchenyang@stu.ahau.edu.cn (C.Z.); 21721689@stu.ahau.edu.cn (F.Z.); 2550361252@stu.ahau.edu.cn (H.L.); chen21720131@stu.ahau.edu.cn (Y.C.); liang2505568381@stu.ahau.edu.cn (L.L.); zchaopu@163.com (C.Z.); isaac777@ahau.edu.cn (W.Z.); liuerbao@ahau.edu.cn (E.L.); shiyingyao@ahau.edu.cn (Y.S.); 2 Kunpeng Institute of Modern Agriculture at Foshan, Foshan 528200, China

**Keywords:** salt tolerance, selection, correlated phenotypic responses, QTL clusters, rice

## Abstract

Salinity is a major factor limiting rice productivity, and developing salt-tolerant (ST) varieties is the most efficient approach. Seventy-eight ST introgression lines (ILs), including nine promising lines with improved ST and yield potential (YP), were developed from four BC_2_F_4_ populations from inter-subspecific crosses between an elite *Geng* (*japonica*) recipient and four *Xian* (*indica*) donors at the Institute of Crop Sciences, Chinese Academy of Agricultural Sciences. Genome-wide characterization of donor introgression identified 35 ST QTLs, 25 of which harbor 38 cloned ST genes as the most likely QTL candidates. Thirty-four are *Xian-Geng* differentiated ones with the donor (*Xian*) alleles associated with ST, suggesting differentiated responses to salt stress were one of the major phenotypic differences between the two subspecies. At least eight ST QTLs and many others affecting yield traits were identified under salt/non-stress conditions. Our results indicated that the *Xian* gene pool contains rich ‘hidden’ genetic variation for developing superior *Geng* varieties with improved ST and YP, which could be efficiently exploited by selective introgression. The developed ST ILs and their genetic information on the donor alleles for ST and yield traits would provide a useful platform for developing superior ST and high-yield *Geng* varieties through breeding by design in the future.

## 1. Introduction

Rice is one of the most important staple crops feeding half the world’s population. It is expected that the world population will continue to grow and exceed nine billion by 2050, which demands a nearly 70% increase in food production [1]. Primarily grown in Asia, rice crops suffer a wide range of abiotic stresses such as salt, submerge, and extreme temperatures [2]. Among these abiotic stresses, soil salinization is an alarming issue, as it affects more than 6% of the agricultural land worldwide, particularly along the coastal areas of rice-growing countries. Furthermore, the threat of salinity to rice production is expected to increase as the sea level rises from global warming [3,4]. The damaging effects of salt stress on rice plants usually include decreased photosynthesis, inhibited plant growth, biomass loss, partial sterility, and thus varied degrees of yield losses [5,6]. Most rice varieties are sensitive to salt, and their sensitivities change with age, most acute at the seedling (2–3 leaf) age, moderately tolerant at the tillering stage, and highly sensitive at the reproductive stage [7,8]. Different rice accessions are known to vary considerably in their salt tolerance (ST). The use of salinity-resistant rice varieties is an important topic, especially in organic farming, with regard to the reduction of synthetic products and chemical fertilizers and to increase product quality, such as the ‘Green Super Rice (GSR)’ which is based on the results of functional genomics research, improving the new varieties with less pesticide and fertilizer, water saving and drought/salt tolerance, high quality and yield were used to realize the fundamental transformation of crop production mode and promote the green development of agriculture [9]. Thus, developing ST rice varieties is the most effective approach to increasing rice productivity, particularly in the coastal areas of Asia.

In the past decades, most breeding efforts for developing ST rice varieties have used relatively few ST accessions as ST donors in their crossing schemes with limited success. This is largely because ST in rice is a quantitative trait controlled by multiple genes and involves complex biological and genetic mechanisms [10,11]. To facilitate the genetic improvement of rice ST, considerable signs of progress have been made in identifying genes and quantitative trait loci (QTLs) controlling ST and its components in rice using bi-parental segregating populations or random germplasm populations in the past two decades, resulting in the identification of hundreds of ST QTLs across the rice genome [11,12]. However, past research on the genetic and molecular dissection of rice ST has been primarily focused on ST at the seedling stage. This is largely because young rice seedlings tend to be more sensitive to salt [13] and partially because it is easier to evaluate rice ST at the seedling stage. Although rice suffers more yield reduction from salt stress at the reproductive stage, phenotyping rice ST at the reproductive stage in genetic and breeding research has been considered to be more time-consuming and laborious [14,15]. More importantly, rice ST at the seedling stage is poorly correlated with its ST at the tillering and reproductive stages, indicating different sets of genes involved in ST at different developmental stages [8,16]. Among the few reported studies on rice ST at the reproductive stage, Mohammadi et al. (2013) reported the identification of 35 QTLs for 11 traits associated with ST at the reproductive stage in an F_2_ population of cross Sadri/FL478, most of which were found to be novel [17]. Mondal et al. (2022) also reported the identification of 40 ST QTLs in a large BC_1_F_2_ population [18]. In both cases, ST QTLs were identified in the F_2_ or BC_1_F_2_ population, in which the phenotypic effects of the ST QTLs were determined based on their associations with yield-related traits under salt stress. Thus, it remains unknown if these QTLs affect the same traits under non-stress conditions, which is of great interest to breeders. *Saltol*, a major QTL on chromosome 1 for ST at the seedling stage [19], is known to be associated with an ST component, the Na^+^/K^+^ ratio, at the reproductive stage [20]. An additional ST QTL gene, SKC1, was reported to function by maintaining K^+^ homeostasis in rice [16,21]. Researchers identified several QTLs on rice chromosomes 1, 7, 8 and 12 for ST at the reproductive stage using a recombinant inbred population. Furthermore, (Takehisa et al., 2004) reported 17 ST QTLs identified in BC_1_F_9_ to BC_1_F_12_ populations of Nipponbare/Kasalath [22]. Two of these QTLs showed strong epistatic effects. Moreover, (Ammar et al., 2009) reported 25 QTLs for 17 traits, including seedling salt-injury score, Na^+^, K^+^, Cl^–^ concentrations and Na^+^/K^+^ ratio in the leaf and stem at the vegetative and reproductive stages using an F_2:3_ population of CSR27/MI48. Meanwhile, considerable progress has been made in the functional genomic research of rice, which has resulted in the cloning and functional characterization of at least 50 genes related to rice ST [1,20]. In contrast, theoretical advances in genome mapping and functional genomic research have not yet been widely utilized in the genetic improvement of rice ST, except for the successful transfer of *Saltol* into some mega-rice varieties by marker-assisted introgression [11], in which FL478 was almost exclusively used as the ST donor. However, the introgression of single large-effect genes/QTLs from a few highly tolerant donors for improving rice ST is not the best choice to resolve the salinity problem, given the large numbers of ST loci involved and possible epistasis among the identified ST loci [23]. Lack of information on the genetic control of rice ST at the reproductive stage also limits our efforts in developing ST rice varieties. Fortunately, an efficient strategy, “selective introgression”, that integrates introgression of useful genes/alleles from diverse germplasm accessions by BC breeding and strong phenotypic selection with marker-assisted tracking and characterization of donor alleles for target and non-target traits in the selected BC progeny has been well-demonstrated for simultaneous improvement and allelic mining of abiotic stresses in rice [24].

We report here the presence of a rich but largely ‘hidden’ genetic variation for rice ST at the vegetative and reproductive stages, yield potential in the rice subspecific gene pools, and demonstrate a powerful strategy to efficiently exploit and dissect this valuable source of genetic variation using the strategy of selective introgression, a complex process involving random introgression of diverse donor segments into an elite genetic background through backcrossing, strong phenotypic selection plus replicated progeny testing to identify promising BC progenies with desirable target traits and genetic tracking of donor segments and gene/QTL identification of selected BC progeny by DNA markers [24].

## 2. Results 

### 2.1. Selection Efficiencies for Improving ST

The salt stress during the initial screening severely hindered the growth and development of the recipient, CY1, and caused a severe reduction in SF (17.1%) and GYP (2.4 g). However, we were able to select 126 BC_2_F_4_ plants which had significantly higher SF (>32.3%) and GYP (>4.7 g) than CY1, from the 2500 plants of the four BC_2_F_4_ populations, including 24 plants from population CY1/Bg90-2, 30 plants from population CY1/X21, 30 plants from population CY1/X22, 29 plants from CY1/Q5, and 13 plants from population CY1/SN265, respectively (Supplementary Table S1).

ANOVA of the data from the replicated progeny testing of the 126 selected BC_2_F_5_ ILs under salt stress and non-stress conditions indicated that the variance components between the salt treatments, among different populations, among ILs within each of the populations and ILs × stress treatment were all highly significant for all measured traits. In the progeny testing, CY1 (check) suffered severe reductions in all yield traits, including 19.1 g (84.5%) reduced GYP, 23.5% (38.3%) reduced SF, 65.0 (56.7%) reduced FGN, 39.7 (29.9%) reduced SNP, 1.6 g (16.1%) reduced TGW, 3.6 (50.0%) reduced PN, 38.8 cm (31.2%) reduced height, and 11.7 days (11.1%) delayed heading. Comparatively, the 126 selected ILs suffered much less with an average 18.5 g (79.0%) reduced GYP, 27.3% (32.5%) reduced SF, 65.0 (49.3%) reduced FGN, 39.7 reduced SNP (25.2%), 1.6 g (6.7%) reduced TGW, 2.8 reduced PN (41.0%), 36.9 cm (27.2%) reduced height, and only three-day (2.9%) delayed heading.

Of the 126 selected BC_2_F_5_ ILs, 80 lines showed significantly *(p* < 0.005) higher GYP than CY1 under salt stress in the replicated progeny testing (Table S2), including 17 ILs from population CY1/Bg90-2 with an average GYP of 10.3 g, 21 ILs from population CY1/X21 with an average GYP of 9.1 g, 18 ILs from population CY1/X22 with an average GYP of 9.1 g, 22 ILs from population CY1/Q5 with an average GYP of 9.4 g, and only two ILs from the intra-subspecific population of CY1/SN265, which out-yielded CY1 by 48.6–194.3% (Table S2). Notably, the selection efficiency for improving ST was 9.75 times higher in the four inter-subspecific populations with *Xian* donors (3.9%) than in the intra-subspecific population (0.4%), indicating that the *Xian* gene pool contained rich useful genetic diversity for improving ST of *Geng* cultivars.

### 2.2. Phenological Mechanisms Underlying Rice ST

When the results of the progeny testing were carefully examined (Table S2), three interesting observations were noted regarding the phenotypic responses in the yield traits of ST ILs to salt stress. First, when compared to CY1, the significantly improved ST (GYP under salt stress) of most ST ILs was associated with significantly higher SF/PH/TGW and less delayed heading under salt stress, though the higher SF/PH and heading delay varied considerably in magnitude among ST ILs from different populations. Secondly, more dramatically reduced SNP was observed in most ST ILs, except those from population CY1/Bg90-2, compared to CY1. Thirdly, ST ILs from population CY1/Q5 showed less reduced SF and PN but were accompanied by more dramatically reduced height under salt stress compared with CY1. These results suggested the presence of different physiological and genetic mechanisms for the improved ST of different ILs. Under salt stress, GYP was positively correlated with PH and its direct components, SNP, PN, and SF, but not with TGW and negatively correlated with HD (Table S3). A positive correlation also existed between PH and SNP and PH and HD, while HD negatively correlated with SF in the ST ILs. Under normal conditions, a positive correlation was observed between GYP and PN or SNP, between HD and PH or PN, while a negative correlation was observed between HD and SF.

### 2.3. Identification and Mapping of ST QTLs

Based on the genotypic data of 210 polymorphic markers, the overall introgression (heterozygosity) in the initially selected 126 BC_2_F_4_ plants was 0.108 (0.013), 0.137 (0.069), and 0.122 (0.027) in populations CY1/Bg90-2, CY1/X21, and CY1/X22, respectively, largely fitting the Mendelian expectations (Table S4), but was 0.216 (0.086) in population CY1/Q5, significantly higher than the Mendelian expectations. To understand the genetic basis of ST in the selected ILs, we adopted the strategy of selective introgression (Zhang et al., 2021 [22]) to detect the donor genomic segments that were strongly associated with the significantly improved ST in the 78 ST ILs from the four inter-subspecific populations. Because only two lines from cross CY1/SN265 were confirmed to have significantly improved ST, this population was not used for genetic mapping because of the low power in QTL detection.

**Figure 1:**
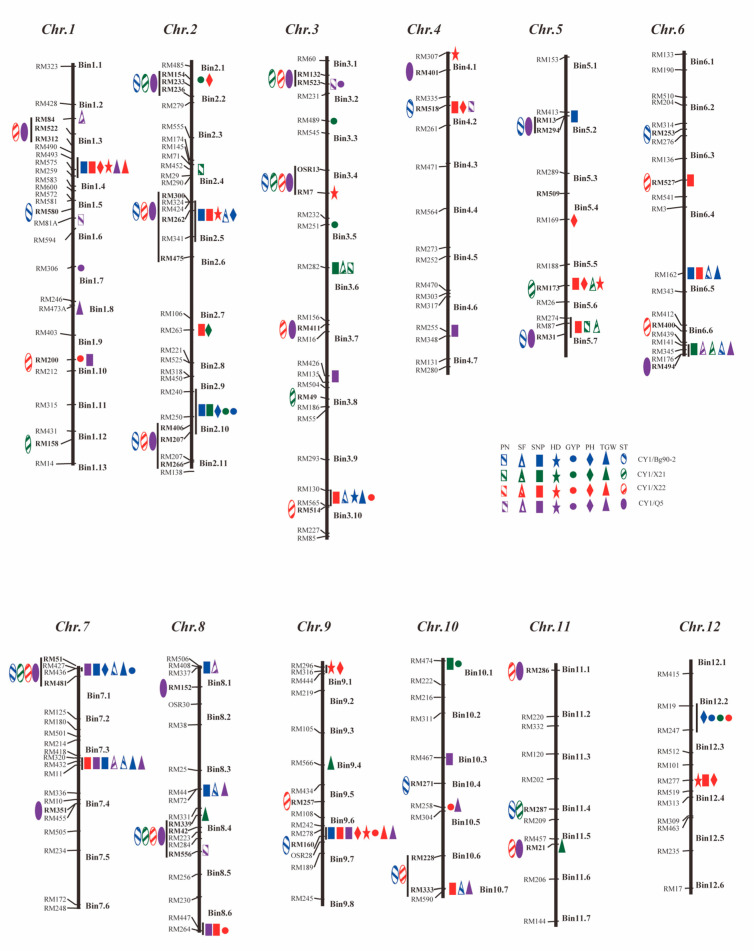
Genomic distribution of 35 loci (on the left side of rice chromosomes) for salt tolerance (ST) and 102 QTLs (on the right side of chromosomes) identified by positive selection in 78 ST introgression lines (ILs) and those ILs selected for high-trait values of seven yield traits from four BC_2_F_4_ populations derived from inter-subspecific crosses between a *Geng* (*japonica*) variety, Chaoyou1 (CY1), and four *Xian* (*indica*) donors. At all these QTLs, the donor alleles were associated with reduced trait values.

Based on a threshold of *p <* 0.001 in the χ^2^ tests against the average whole genome introgression of respective populations, we were able to detect 35 ST QTLs on 11 rice chromosomes, except for chromosome 12 (Figure 1 and Supplementary Table S4). These included 15 ST QTLs from population CY1/Bg90-2, which showed an average donor introgression of 0.483, 4.5 times as much as the whole genome introgression of 0.108; nine ST QTLs from population CY1/X21, which showed an average donor introgression of 0.465, 3.4 times as much as the whole genome introgression of 0.137; 17 ST QTLs from population CY1/X22 which showed an average introgression of 0.482 or four times as much as the whole genome introgression of 0.122; and 17 ST QTLs from population CY1/Q5 which showed an average introgression of 0.494 or 2.3 times as much as the whole genome introgression of 0.216. Important ST QTLs included *qST3.4* and *qST7.1,* which were detected in all four populations, plus *qST2.5* and *qST3.2,* which were detected in three populations. Thirteen additional ST QTLs were detected in two of the four populations, while the remaining 17 were detected in a single population.

**Figure 2:**
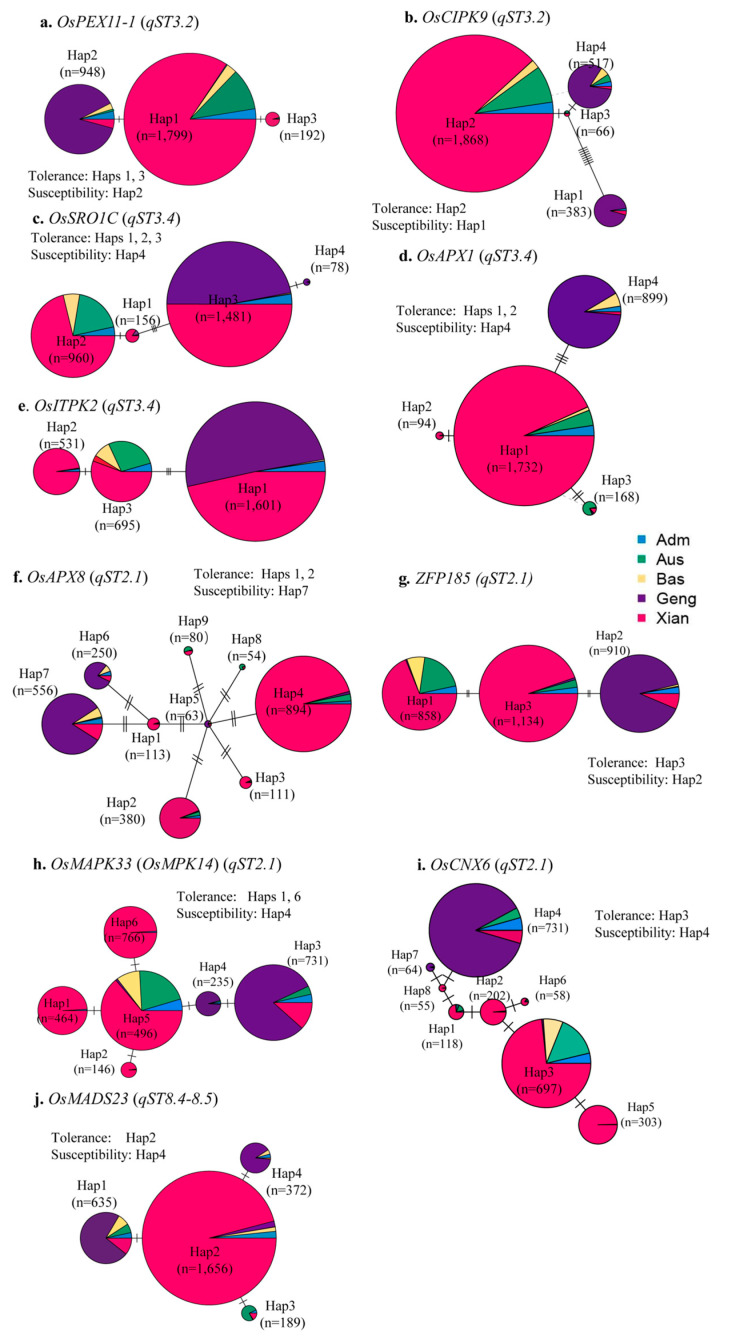
Haplotype networks of 10 salt tolerance genes co-localized with four ST QTLs, the frequency distributions of the major alleles (gcHaps) at each of the loci in five major rice populations (Adm, Aus, Bas, Geng, and Xian) of the 3000 rice core collections [26], and the associations of major alleles (gcHaps) at each of the loci with salt tolerance or susceptibility detected in ST introgression lines from four BC_2_ populations (Tables S4 and S7); (**a**–**j**) subfigures represent genes.

We searched the genomic regions 5 cM flanking each (the peak marker) of the identified ST QTL regions and found that 25 of the 35 ST QTL regions harbor 38 cloned genes reported to be associated with ST and/or other abiotic stress tolerances (Tables S4 and S7). Clearly, these are potential candidate genes for the identified ST QTLs. To find additional evidence supporting these genes as candidates for the identified ST QTLs, we performed comprehensive gene CDS haplotype (gcHap) analyses for 31 ST genes in the 3000 diverse rice accessions (Wang et al. 2018 [25]) and the five parents (Figure 2 and Figure S2). Table S7 shows the gcHap diversity (*E_H_*) and the number of major gcHaps (gcHapN) in different rice populations and the parents, which varied considerably for different ST genes. Of these ST genes, only four genes (*DSM3*, *OsCIPK15, OsHDT1*, and *OsCIPK15*) are conserved ones with relatively low *E_H_* and gcHaoN, while the remaining 34 ST genes are all of *Xian*-*Geng* differentiated type with each subspecies having one predominant (allelic frequency >70%) but different gcHap. For 31 of the ST loci with the parental gcHap information, the recipient (CY1) and donors have different gcHaps at 28 ST genes, except for *SNAC3*, *OsMYB48-1,* and *OsAKT2*. At all 28 ST gene loci, the donor (*Xian*) gcHaps were associated with ST, while the CY1 (*Geng*) alleles were associated with salt susceptibility. However, it was noted that in 17 of these cases, the same donor alleles (gcHaps) at the ST genes were not detectable in all populations, even though >2 of the donors had the same gcHaps. For example, all four donors have Hap3 at *OsHAK5,* but it was detected as an ST QTL only in CY1/X21. In nine cases (*OsMAPK33*, *OsAPX8*, *GnT1*, *OsPEX11-1*, *OsSRO1C*, *OsAPX1*, *OsHAK16*, *qSE3*, and *OsWRKY08*), more than one donor gcHap was associated with ST.

Figure 2 shows the haplotype networks of the nine most likely candidate genes for four important ST QTLs identified in ≥3 of the BC populations. For *qST3.2*, we had two candidates, *OsPEX11-1* and *OsCIPK9*. *OsPEX11-1* encodes a downstream gene in the ABA-mediated pathway for enhanced ST in rice [27]. Our gcHap analyses identified three major gcHaps at *OsPEX11-1* in rice populations, with Hap2 predominant in subspecies *Geng* and CY1 as the susceptibility allele. At the same time, Hap1 and Hap3 were predominant in subspecies *Xian* as the candidates for *qST2.1* detected in all four *Xian* donors (Figure 2a, Supplementary Table S7). *OsCIPK9* encodes calcineurin B-like protein-interacting protein kinase-9, whose non-functional mutant has enhanced ST [28]. Four major gcHaps at *OsCIPK9* are present in rice populations. CY1 carries the susceptibility Hap4 predominant in subspecies *Geng*. Differing from Hap4 by four and five synonymous mutations in the *OsCIPK9* CDS region, Hap1 and Hap2 were predominant in subspecies *Xian* and detected as the candidates for *qST2.1* in the four *Xian* donors (Supplementary Table S7). A detailed comparison between Hap4 and Haps1, 2, and 3 indicated these nonsynonymous mutations occur at exon12 of the gene, causing aa substitutions from Tyrosine to Histidine in exon12 (Figure 1).

For *qST3.4*, we identified two candidate genes, *OsSRO1C* and *OsAPX1*. *OsSRO1C* encodes a rice SRO protein regulated by *SNAC1,* and the loss-of-function mutant of *OsSRO1C* showed increased stomatal aperture and sensitivity to drought [29]. In contrast, *OsAPX1* encodes a rice cytoplasm ascorbate peroxidase, which, when transformed into Tobacco plants, causes enhanced ST [30]. We identified four major gcHaps at *OsSRO1C,* with Hap2 present only in population *Xian* and Hap4 only in population *Geng*. Haps 1, 2 and 3 were detected as ST QTLs in the four donors, while Hap4 was associated with salt susceptibility in CY1 (Figure 2c). Similarly, four major gcHaps were identified at *OsAPX1,* with Hap1 and Hap2 predominant in subspecies *Xian,* while Hap3 is present only in populations *Aus* and *Xian* for ST. Carried by CY1 for salt susceptibility and predominant in subspecies *Geng*, Hap4 differs from Hap1, Hap2 and Hap3 by three, four and five synonymous mutations of exons 7 on the *OsAPX1* CDS region (Figure 2d), which cause an amino acid (aa) substitution from Arginine to Cysteine in exon7 (Figure 1).

We identified four ST genes, *OsAPX8*, *ZFP185*, *OsMAPK33* (*OsMPK14*), and *OsCNX6*, in the region of *qST2.1* (Figure 1, Supplementary Table S4). *OsAPX8* encodes a chloroplast ascorbate peroxidase, and reduced expression of *OsAPX8* leads to enhanced ST in rice [31]. Eight major gcHaps at *OsAPX8* are present in rice populations (Figure 2e). CY1 carries Hap7, which is present in most *Geng* accessions, while Haps 1 and 2 at *OsAPX8* were candidates for *qST2.1* detected in three donors (Q5, X21, and X22) (Supplementary Table S7). ST Haps 1 and 2 were present in only 493 *Xian* and *Aus* accessions and differed from the susceptibility Hap7 by two and six nonsynonymous mutations in the *OsAPX8* CDS region. *ZFP185* encodes a C2H2 zinc-finger protein which interacts with *OsMAPK3* to enhance salt tolerance in rice [27]. We found three major gcHaps at *ZFP185* in rice populations, with Hap2 predominant in subspecies *Geng* and CY1 for salt susceptibility. Differing from Hap2 by only two nonsynonymous mutations in the gene CDS region, Hap3 is predominant in subspecies *Xian* and one candidate of *qST2.1* detected in donors Q5, X21, and X22 (Figure 2f, Supplementary Table S7). *OsMAPK33* encodes *a* mitogen-activated protein kinase whose overexpression leads to enhanced rice sensitivity to salt [32]. Six major gcHaps are present at *OsMAPK33* in rice populations, with Hap3 and Hap4 predominant in subspecies *Geng*, while Hap1, Hap 5, and Hap 6 are predominant in subspecies *Xian* (Figure 2f, Supplementary Table S7). As one candidate for *qST2.1*, ST alleles Hap1 and Hap6 in donors Q5, X21, and X22 differ from the salt susceptibility CY1 Hap4 by only two nonsynonymous mutations in the *OsMAPK33* CDS region. Encoding homologs of *MoaE* and *MoeA* for ST in rice [33], *OsCNX6* has eight major gcHaps in rice populations, with Hap3 predominant in subspecies *Xian* and Hap4 predominant in subspecies *Geng*. As one candidate for *qST2.1*, ST Hap3 in donors Q5, X21, and X22 differs from the susceptibility CY1 Hap4 by four nonsynonymous mutations in the *OsCNX6* CDS region.

Only a single candidate ST gene, *OsMADS23*, was found in the *qST8.4-8.5* region. *OsMADS23* is a MADS-box transcription factor gene reportedly to confer rice osmotic stress tolerance by regulating ABA biosynthesis [34]. With four major gcHaps at *OsMADS23* in rice populations (Figure 2f), the tolerance, Hap2, was predominant in subspecies *Xian*. Differing from Hap2 by only a single nonsynonymous mutation, the susceptibility Hap4 was present in more than 350 *Geng* accessions. Interestingly, Hap3 was predominant only in the population *Aus*, but it remains unknown for its effect on ST. Taken together, these results strongly suggested that the donor gcHaps at many of these ST genes were most likely to be the candidate genes for the identified ST QTLs.

### 2.4. Correlated Phenotypic Responses from Selection for ST and Mapping of QTLs for ‘Non-Target’ Traits

Under non-stress conditions of the progeny testing, selection for ST (GYP under salt stress) resulted in correlated responses of significantly increased GYP in 40 (51.3%) of the 78 ST ILs from the four populations by an average of 8.5 g (37.6%) over CY1 under normal conditions. Most of the correlated responses in GYP could be attributed to significantly increased SNP in 57 (73.1%) of the ST ILs, with an average SNP of 44.9 (33.5%) over CY1. This was in contrast to only eight ST ILs that showed significantly reduced GYP by an average of 7.0 g (31.0%), six of which could be attributed to significantly reduced SF under normal conditions. Similarly, correlated responses for significantly increased PH by 19.6 cm occurred in 53 (74.6%) of the ST ILs from populations CY1/X21, CY1/X22, and CY1/Q56, in contrast to only 5 (7.0%) ST ILs showing significantly reduced average PH by 10.7 cm under normal conditions. In 35 of these cases, the correlated increases in PH were associated with delayed heading (Supplementary Table S2). In the same three populations, the correlated responses by an average 7.6-day delayed heading were observed in the 48 (67.6%) ST ILs, in contrast to only 3 (4.2%) ST ILs showing significantly early heading by 4.3 days under normal conditions. Taken together, we were able to identify nine promising ILs which produced significantly higher GYP under both salt stress and non-stress normal conditions with very small changes in PH and HD (Table 1). The progeny testing identified the two best lines, A-12 and C-30, from populations CY1/Bg90-2 and CY1/X22. With yield increases of 211.4% and 77.4% under salt stress and normal conditions, line A-12 had significant improvements in all yield traits except for slightly reduced TGW by 1.7 g, plus 6.6 cm reduced height and three days of delayed heading as compared to CY1 under normal conditions. With yield increases of 297.1% and 50.9% under salt stress and normal conditions, line C-30 had significant improvements in all yield traits except for slightly reduced SF of 4.4%, 18.6 cm shorter and two days of early heading compared to CY1 (Table 1).

### 2.5. Identification and Mapping of QTLs Affecting the Yield Traits under Normal Conditions

The significant amounts of residual genetic variation for the measured yield traits in the 113 ILs initially selected from the four populations under non-stress normal conditions in the progeny testing allowed us to select ILs showing significantly (*p* < 0.01) lower (negative selection) or higher (positive selection) trait values than CY1 for each of the measured traits. These included 11 low GYP ILs and 17 high GYP ILs from all four populations, eight low SF ILs from populations CY1/X22 and CY1/Q5, 11 high SF ILs from populations CY1/Bg90-2, CY1/X21, and CY1/Q5, 32 high SNP ILs from all four populations, 13 low-PN ILs populations CY1/Bg90-2, CY1/X21, and CY1/Q5, five high PN ILs from populations CY1/X21 and CY1/Q5, 11 low TGW ILs from populations CY1/Bg90-2, CY1/X21, and CY1/X22, 15 high TGW ILs from all four populations, seven low PH ILs from populations CY1/Bg90-2 and CY1/X22, 67 high-PH ILs from all four populations, six low HD ILs and 11 high HD ILs from populations CY1/Bg90-2 and CY1/X22, respectively. Using these low- and high-trait value ILs and the approach of selective introgression (Supplementary Table S4), we were able to identify and map 160 QTLs responsible for the associated responses in the seven yield traits in the selected ILs. These QTLs were mapped to 49 genomic regions across all 12 rice chromosomes, including 102 QTLs in 43 genomic regions with the donor alleles associated with increased trait values detected by positive selection and 58 QTLs in 35 genomic regions with the donor alleles associated with reduced trait values detected by negative selection (Figure 1 and Supplementary Table S5). The number of identified QTLs was 50 in population CY1/Bg90-2, 74 in population CY1/X21, 69 in population CY1/X22, and 41 in population CY1/Q5 (Figure 1; Supplementary Table S5 and Supplementary Figure S1). Nineteen of these QTLs were identified in 2–3 of the populations.

QTLs affecting GYP: Twenty-three QTLs affecting GYP were identified and mapped to 10 rice chromosomes except for chromosomes 4 and 11 (Supplementary Table S5). These included eight QTLs detected in 11 low GYP ILs with the donor alleles causing low GYP (negative selection) and 15 QTLs in 17 high GYP ILs with the donor alleles causing high GYP (positive selection) from the four populations. Of these GYP QTLs, qGY12.2^P^ was detected in three populations, qGY1.4^N^ and qGY7.1^P^ were detected in two populations, and the remaining 22 GYP QTLs were detected in one of the populations.

QTLs affecting SF: Seventeen QTLs affecting SF were identified and mapped to nine rice chromosomes except for chromosomes 9, 11 and 12 (Supplementary Table S5). These included four QTLs detected in eight low SF ILs from populations CY1/X22 and CY1/Q5, with the donor alleles causing low SF (negative selection) and 13 QTLs in 11 high SF ILs with the donor alleles causing high SF (positive selection) from three of the four populations except for CY1/X22. Of these QTLs, qSFY6.6^P^ was detected in three populations, and qSF3.2^N^ and qGY7.3^P^ were detected in two populations. The remaining 14 QTLs were detected in one of the populations.

QTLs affecting SNP: Twenty-eight QTLs affecting SNP were identified and mapped to 11 of the 12 rice chromosomes except for chromosome 11, all of which were detected in 32 high-SNP ILs from the four populations with the donor alleles increasing SNP (positive selection) at all 29 loci (Supplementary Table S5). Of these QTLs, qSNP7.3^P^ and qSNP9.6^P^ were detected in three populations. Five additional QTLs, qSNP1.4^P^, qSNP2.5^P^, qSNP6.5^P^, qSNP7.1^P^, and qSNP8.6^P^, were detected in two of the populations, while the remaining 21 SNP QTLs were detected in one of the populations.

QTLs affecting PN: Twenty-one QTLs affecting PN were identified and mapped to 11 rice chromosomes except for chromosome 11 (Supplementary Table S5). These included 14 QTLs detected in 13 low PN ILs from populations CY1/Bg90-2, CY1/X22, and CY1/Q5 with the donor alleles causing reduced PN (negative selection) and seven QTLs in five high PN ILs with the donor alleles causing increased PN in populations CY1/X21 and CY1/Q5. In addition, two QTLs, qPN5.7^N^ and qPN10.7^N^, were detected in two populations, while the remaining 19 PN QTLs were identified in one.

QTLs affecting TGW: Thirty QTLs affecting TGW were identified and mapped to the 12 rice chromosomes, including 16 QTLs detected in 11 low TGW ILs from populations CY1/Bg90-2, CY1/X21, and CY1/X22 plus 14 QTLs detected in 15 high TGW ILs from the four populations with the donor alleles increasing TGW (Supplementary Table S5). In addition, four QTLs, qGW1.4^P^, qGW7.3^P^, and qGW9.6^P^, were detected in two populations, while the remaining 27 TGW QTLs were detected in one.

QTLs affecting PH and HD: Twenty-four QTLs affecting PH were identified and mapped to 11 of the 12 rice chromosomes except for chromosome 11, including 10 QTLs detected in seven short ILs (negative selection) plus 15 QTLs detected in 14 tall (positive selection) ILs from populations CY1/Bg90-2 and CY1/X22 (Supplementary Table S5). In addition, 57 of the 59 ST ILs from populations CY1/X21 and CY1/Q5 showed significantly increased PH. Thus all 26 ST QTLs (nine in population CY1/X21 and 17 in population CY1/Q5) showed significant over-introgression in the 57 tall ILs. Of these ST/PH QTLs identified in populations CY1/X21 and CY1/Q5, five (qPH2.1^P^, qPH2.5^P^, qPH2.10^P^, qPH5.6^P^, and qPH7.1^P^) were also detected in populations CY1/Bg90-2 and/or CY1/X22. Fifteen QTLs affecting HD were identified and mapped to nine of the 12 rice chromosomes except for chromosomes 6, 7 and 11, including six HD QTLs detected in six early heading ILs (negative selection) plus nine QTLs detected in 11 late heading (positive selection) ILs from populations CY1/Bg90-2 and CY1/X22 (Supplementary Table S5). All these HD QTLs were detected in either population CY1/Bg90-2 or population CY1/X22.

Taken together, several observations were noted regarding the genetic bases of ST and non-target yield traits identified under normal conditions, as summarized in (Supplementary Table S6). First, ILs with increased trait values were found in 158 cases (75.9% of which also showed confirmed ST), resulting in the identification of 136 QTLs at which the donor alleles were associated with increased values of the yield traits under normal conditions. In contrast, ILs with reduced trait values were found in 56 cases (53.6% of which showed confirmed ST), from which the donor alleles were associated with reduced values of the yield traits at 64 QTLs under normal conditions. Second, PN was the only exception in which most ST ILs showed unchanged or fewer PN, with few PN QTLs detected.

It is well-known that the responses of rice plants to salinity vary at different developmental stages, but salt stress causes more severe yield losses at the reproductive stage [35]. Most previous studies focused on rice ST at either the germination and/or seedling stages, primarily because of the difficulty in phenotyping ST at the vegetative and reproductive stages. Meanwhile, breeding progress for improved ST at the reproductive stage has been slow largely because of relatively few germplasm accessions showing high levels of ST. In this study, we tried to improve the ST of CY1, a high-yield commercial but salt-sensitive *Geng* variety, using BC breeding. We started the salt stress at the tillering stage and continued until maturity in both the initial screening and progeny testing because this type of salt stress represented the real cases of most coastal saline areas in Asia where transplanted rice is the predominant way for growing rice crops. Following the previous success in improving rice tolerances to abiotic stresses such as drought, submergence, anaerobic germination, salinity at the seedling stage, heat and cold [36,37,38,39,40,41,42], we tried to test the hypothesis for the existence of rich genetic variation for ST in different sub-specific gene pools of rice. As expected, the development of 78 ST ILs and nine promising lines with greatly improved ST and yield potential, plus the identification of more than 200 QTLs underlying ST and yield traits achieved in this study, again demonstrated the power and efficiency of selective introgression for simultaneous improvement and genetic dissection of multiple complex traits. Specifically, our results revealed several phenological and genetic mechanisms underlying rice ST at the vegetative and reproductive stages that merit further discussion.

## 3. Discussion

### 3.1. The Phenological Mechanisms of Rice ST at the Vegetative and Reproductive Stages

Our results suggest that ST at the vegetative and reproductive stages involved complex phenological and genetic mechanisms, evidenced by the following observations. First, the improved ST of most ILs were reflected by their significantly higher PH/PN and SF/TGW than CY1 under salt stress. The former two traits indicated that these ST ILs were able to maintain relatively normal growth/development at the tillering stage under salt stress. In comparison, the latter two traits indicated that most ST ILs had relatively normal fertilization and grain filling at the reproductive stage [43,44]. Second, most ST ILs showed accelerated heading under salt stress, as compared with CY1, suggesting the possible presence of salt escape [45]. This was because the level of salt stress in the concrete ponds increased with plant development. This was due to continuous irrigation with salty water and water evaporation during plant growth. Thus, early maturing lines were expected to suffer less from salt stress. Thirdly, most ST ILs showed dramatically increased height under both salt stress and non-stress conditions, which suggested the presence of two additional ST mechanisms. One was the possible salt dilution [46] because tall ILs from populations CY1/Bg90-2, CY1/X21, and CY1/X22 had larger plant sizes under both stress and non-stress conditions, even though they showed the same height reduction under salt stress as CY1 (Supplementary Table S2). The other was reflected by the more dramatically reduced height of most tall ILs from population CY1/Q5 under salt as compared with the non-stress conditions, suggesting these ILs might have benefited from a possible ST mechanism by salt compartment in which more salt was deposited in the vegetative parts (leaves and stems) than in the reproductive parts (spikelets and grains) in addition to salt dilution. Fourthly, we observed significantly increased SNP in 32 (41%) of the ST ILs from all four populations; most also showed significantly increased height and GYP under normal conditions. In other words, improved ST in these ILs would have been achieved through their significantly increased SNP and GYP under normal conditions. This would obviously be the preferred ST mechanism by plant breeders because most of these ILs also had significantly improved yield potential under non-stress conditions. Of course, one may perceive that many ST ILs have more than one of the above-mentioned ST mechanisms [47].

### 3.2. Genetic Basis of Rice ST and Its Associated Yield Traits

The most important discovery of this study was the identification of 35 ST QTLs in each of which the donor (*Xian*) allele was associated with significantly improved ST in the selected ILs. These ST QTLs could be categorized into two major groups. The first group involved 25 ST QTLs, each of *which* one or more candidate genes with confirmed functions on ST were found. Thus, many of the identified ST QTLs, particularly those important ones detected in multiple populations (Figure 2), may each have resulted from the accumulated effects of multiple ST genes. Notably, 34 (90%) of the candidate ST genes showed the maximum *Xian*-*Geng* subspecific differentiation with one allele (gcHap) predominant in one subspecies and a different one in the other. In fact, approximately one-fifth of all rice genes belong to this type of *Xian*-*Geng* subspecific differentiated gene [24]. More importantly, at virtually all candidate genes with confirmed functions on ST, it is the CY1 (*Geng*) alleles that were associated with salt susceptibility, while the donor *Xian* alleles were associated with ST, consistent with the QTLs results. Moreover, the differences between the tolerance *Xian* and susceptibility *Geng* alleles involved only one to a few aa substitutions. Thus, the current knowledge that the *Xian*-*Geng* subspecific differentiation of rice is primarily reflected by their respective adaptations to different temperature and water environments is incomplete. In other words, the gradual adaptation of wild and ancient *Geng*-type accessions to drier and colder environments was also accompanied by reduced ST. Then, an important question arises regarding how such small allelic variation involving few *aa* substitutions between the *Xian* and *Geng* alleles at the ST gene loci would cause significant phenotypic differences in ST, which should be addressed in the future.

The second group involved QTL clusters at bins 1.4, 2.1, 2.5, 2.10, 5.7, 6.6, 7.1, and 9.6, at each of which the donor alleles for improved ST were also associated with increased GYP, SNP, SF and PH, responsible for the correlated responses to grain yield traits observed in many of the selected ST ILs. As discussed above, the donor alleles at these QTLs should be more important because their contributions to ST were achieved by improved growth rate and yield under salt. Still, they also contributed to improved yield under non-stress conditions. Empirical evidence from past rice QTL cloning efforts suggests that these QTL clusters with large effects on multiple related traits tend to harbor important regulatory genes, which should be the targets in future rice functional genomics research.

Another important result of this study was the identification of an additional 160 QTLs for seven yield traits in the ST ILs under non-stress conditions, including 58 (in 65 cases) QTLs with the donor alleles for reduced trait values detected by negative selection and 102 QTLs (in 119 cases) with the donor alleles for increased trait values detected by positive selection (Figure 1 and Supplementary Table S5). In this study, at least three factors could have contributed to the high power and efficiency in identifying QTLs affecting complex traits. First, the 500 individuals in the original BC_2_F_4_ populations from sub-specific introgression contained very large genetic variation for ST and the measured yield traits. Thus, the 113 ILs selected from the initial salt screening of 2000 BC_2_F_4_ plants were still segregating at large numbers of loci for the seven yield traits. Notably, most of the identified QTLs were clustered in 31 genomic regions (including 160 QTL clusters affecting ST and yield traits), each of which affected three or more of the yield traits, including several large QTL clusters in bins 1.4, 2.10–2.11, 3.10, 5.6–5.7, 6.6, 7.1, 7.3, 9.6, and 10.7, each of which was associated with >5 of the yield traits (Figure 1; Supplementary Table S5 and Supplementary Figure S1). Genetic pleiotropy was suggested to be responsible for QTL clusters at bins 1.4, 2.9, 2.10, 2.11, 3.2, 3.6, 3.10, 7.1, 7.3, 8.6, 9.6, and 12.2, each of which a GYP QTL was detected with QTLs for one or more of its components (PN, SNP, SF, and TGW) by the same marker in the same population, and in eight additional cases where a QTL for reduced height co-localized with a HD QTL for early heading, or vice versa in the same population. Possible physiological pleiotropy was suggested in seven cases of negative associations between QTLs for yield components (SNP, SF, and TGW) at bins 1.4, 2.7, 3.6, 3.8, 4.2, 5.6, and 12.4 identified in the same populations. The linkage could be responsible for the majority of the remaining detected QTL clusters. Among the QTL clusters identified, we noted six cases (*qPN2.4^P^* and *qPN2.4^N^*, *qPH2.5^P^* and *qPH2.5^N^*, *qGW3.10^P^* and *qGW3.10^N^*, *qPN5.7^P^* and *qPN5.7^N^*, *qSF6.6^P^* and *qSF6.6^N^*, and *qGW3.10^P^* and *qGW3.10^N^*) at each of which, QTLs affecting the same traits were detected by both positive and negative selection with the same or tightly linked markers but most in different populations (Supplementary Table S5 and Supplementary Figure S1). Genetically, this could be due to linkage drag or the possible presence of multiple alleles from different donors at the same QTLs. We believe linkage drag could be more likely responsible for most of these cases because the presence of linkage drag in the *Xian* genomes from the tight repulsive linkage of QTLs with opposite effects on rice yield traits could be relatively easily detected in the same population by positive and negative selection [2,48,49].

### 3.3. Implications for Improving Rice ST at The Vegetative and Reproductive Stages

Our results have important implications in breeding for improved ST at the vegetative and reproductive stages. First, all five donors in our BC breeding populations were sensitive to salt. Still, many more ST BC progeny were identified in the four populations with *Xian* donors than the one with *Geng* donors (SN265), indicating that the *Xian* gene pool contains rich, valuable genetic variation for improving ST, and much of this variation is ‘hidden’ because none of the donors had high levels of ST. Similar results were obtained in previous efforts to improve tolerances to cold, heat and drought [38,39,40,41]. All these results indicate that the subspecific gene pools, *Xian* or *Geng*, contain rich, valuable genetic variation not only for improving abiotic stress tolerances but also for improving yield potential for each other. Exploiting this rich ‘hidden’ diversity can be easily achieved by subspecific introgression through BC breeding. Notably, different donors did differ in their contributing genetic, physio- and morphological mechanisms underlying ST and yield traits, which could readily be discovered and exploited through selective introgression but not through phenotyping the parental lines. Secondly, strong phenotypic selection in the initial screening for ST using SF as the primary target trait followed by replicated progeny testing under both salt stress and non-stress conditions using yield as the target trait was powerful and essential for simultaneous improvement of ST and yield potential of rice. Moreover, large segregating breeding populations should be used in the first round of selection for ST to ensure a high selection efficiency for improving ST and yield potential, to break the possible genetic drag, to take advantage of correlated responses for increased yield traits and to overcome hybrid breakdown commonly observed in most segregating populations from inter-subspecific crosses of rice [48]. This was consistent with previously reported breeding efforts for improving tolerances to drought, cold and heat [35,36,38,39,40,41,47]. Finally, the development of nine promising lines with short stature, early heading, large SNP and GYP, in addition to excellent ST (Table 1), strongly suggested that the undesirable phenotypic association between ST and high PH and/or delayed heading observed in this study could be broken, even though it remains unknown whether tight linkage or genetic pleiotropy was the underlying genetic basis for the association. More importantly, the developed ST ILs plus their genetic/phenological constitutions in ST and yield traits provide useful information for breeders to develop better varieties in the future using novel breeding strategies such as breeding by design or designed QTL pyramiding [24,49,50,51,52].

## 4. Materials and Methods

### 4.1. Plant Materials

The materials used in this study included an elite *Geng* (*japonica*) variety, Chaoyou 1 (CY1) used as the recipient, four *Xian* (*indica*) donors from Vietnam (X21, X22, and Q5) and Sri Lanka (Bg90-2), and one *Geng* donor, Liao*Geng*265 from Northeast China. Crosses were made between CY1 and the donors to create F_1_ plants, which were then backcrossed to CY1 to produce BC_1_F_1_ plants. Then, approximately 25 BC_1_F_1_ plants of each cross were backcrossed to CY1 to produce 25 BC_2_F_1_ lines. Selfed seeds from all ~25 BC_2_F_1_ lines were bulk harvested to produce a bulk BC_2_F_2_ population. This selfing process (without selection) continued for two more generations for all BC populations until large amounts of BC_2_F_4_ seeds were obtained for each BC population (Table 1).

### 4.2. Screening BC_2_F_4_ Progeny for Salt Tolerance at the Vegetative and Reproductive Stages

In the normal rice growing season (May to October), seeds of the BC_2_F_4_ segregating populations were sown in the seedling nursery, and 500 seedlings (30-day-old) of each BC_2_F_4_ population were transplanted into the concrete tanks (3 × 6 m) at a spacing of 15 × 20 cm with two rows of CY1 as the check in each tank in the screening house at the Rice Research Institute, Tianjin Academy of Agricultural Sciences (TAAS). The transplanted seedlings were allowed to grow under normal freshwater irrigation for 15 days. For the salt treatment, we mixed approximately equal amounts of salt water from two wells with a water salt (NaCl) content of 1.6% in one well and 0.3% in the other. The salt content of the mixed water was adjusted to ~0.8% (~140 mmol·L^−1^ NaCl). Then, the mixed water was used to irrigate all the concrete tanks every 3–5 days from 15 days after transplanting until maturity. Under this type of salt treatment, the soil salt level in the ponds was expected to increase slowly from evaporation during the growth period of rice plants. However, this increase varied over time from outside weather conditions, which resembled the salt stress in most natural conditions and thus met our primary purpose in developing salt-tolerant rice varieties and identifying genes/QTLs for salt tolerance. At maturity, a total of 126 BC_2_F_4_ plants showing significantly higher seed setting and yield than CY1 (the susceptible recurrent parent) were visually selected for measuring panicle number (PN), spikelet and filled grain numbers per panicle (SNP and FGN), and grain yield per plant (GYP, g). The number of selected salt-tolerant (ST) plants ranged from 13 from population CY1/SN265 to 50 from population CY1/X21 (Table 1).

The progeny testing of the selected 126 BC_2_F_5_ lines was conducted in the next normal season (May–Oct) under the same stress conditions in TAAS and normal non-stress conditions in the experimental farm of Institute of Crop Sciences, Chinese Academy of Agricultural Sciences. Under both experiments under salt stress and normal conditions, seeds of the 126 BC_2_F_5_ lines were sown in the seedling nurseries, and 30-day-old seedlings of each line were transplanted into a single-row plot with 11 plants in each plot at a spacing of 15 × 20 cm. The plots were arranged in a complete randomized block design with two replications for each of the selected ST lines. Four plots of CY1 were randomly inserted into the experiment under stress and non-stress conditions. The field arrangements and management of the experiments were the same for the stress and non-stress conditions except for the salt treatment, which was the same as that for the initial salt treatment in the previous season described above. Five representative plants in each plot were measured for heading date (days) and plant height (cm) in the field. At maturity, three representative plants from each plot were harvested and dried in an oven and then measured for PN, FGN, SNP, spikelet fertility (SF% = FGN/SNP × 100), 1000-grain weight (TGW, g), and GYP.

### 4.3. The Genotyping Experiment

Bulked fresh leaf tissues from each BC_2_F_4_ ILs were collected from the pooled leaf tissues of individual BC_2_F_4_ ILs from the seedling nursery. DNA was extracted using the CTAB method [53]. The parental polymorphism was surveyed using more than 600 common single sequence repeat (SSR) microsatellite markers (https://archive.gramene.org/markers/), and 210 evenly distributed polymorphic SSR markers were used for genotyping the 126 BC_2_F_5_ lines initially selected from the salt screening.

### 4.4. Statistical Analyses

ANOVA was used to summarize the variation of each trait among different ILs (G) between the stress treatments (S) and G × S interactions using the R software (http://www.r-project.org/). The R software was used for correlation analysis of different traits under the same or different treatments.

### 4.5. Identifying QTLs Affecting ST and Yield Traits under Non-Stress Normal Conditions

QTLs affecting ST (target trait) and non-target traits measured under non-stress conditions were identified using the data from replicated progeny testing described above and the strategy of selective introgression [24,48]. Specifically, for identifying ST QTLs, the replicated progeny testing confirmed that 78 of the ILs initially selected from four populations with *Xian* donors showed significantly (*p* < 0.01) higher GYP under salt stress, which were used for identifying ST QTLs. According to the population genetics theory [54,55], the confirmed ST ILs were expected to show significant over-introgression at loci associated with ST. Thus, χ^2^ tests were performed to scan the whole genome to identify marker loci that showed significant (*p* < 0.001) deviation in genotypic frequencies in the ST ILs of a specific population from the whole genome donor introgression of the population, assuming that at most segregating loci in the genome, the donor alleles were not associated with ST and thus could be used as the random expectation for the χ^2^ tests.

To identify QTLs affecting the seven yield traits, we first select ILs with significantly (*p* < 0.01) higher or lower trait values than CY1 for the seven yield traits under non-stress conditions of the progeny testing. Then, χ^2^ tests were performed to scan the whole genome to identify marker loci that showed significant *(p* < 0.001) deviation in genotypic frequencies in the low- and high-trait value ILs. Because selection was also expected to result in strong non-random associations between or among loci contributing to the selected traits [56], we performed the multi-locus independence probability tests using the genotypic data of selected high- or low-trait value ILs to identify association groups (AGs), each consisting of a group of unlinked loci (markers) but perfectly associated loci in a set of high- (GYP, SNP, and PH) or low-trait (TGW) value ILs. The multi-locus independence probability was obtained by the formula, $P_{\left(AG\right)}={\left(p_{i}\right)}^{rm}•{\left(1-p_{i}\right)}^{r\left(n-m\right)}$, where *p_(AG)_* is the probability of a group of *r* (*r* ≥ 2) unlinked loci that are perfectly associated with one another in ILs selected for high- or low-trait value ILs, $p_{i}$ is the expected frequencies of the donor introgression at *r*th locus in the selected ILs (*i* = 1, 2, 3 … *r*), n is the number of ILs, m is the number of the ILs that have co-introgression of the donor alleles at the r unlinked loci, and $\left(n-m\right)$ is the number of ILs having no introgression at the r unlinked loci in the AG. Theoretically, an AG could be a group of loci acting epistatically in the positively regulating signaling pathway affecting the selected trait segregating in the ILs chosen [56]. Because very few ILs with significantly higher or lower trait values than CY1, we adopted double criteria to claim an AG associated with the selected traits, i.e., the donor introgression frequency, F_(donor)_, at each of the loci in an AG was significantly higher than the whole population introgression (*p* < 0.05) based on the χ^2^ test and *p_(AG)_* < 0.0001 to minimize the false negative probability.

### 4.6. Candidate Genes for the ST QTLs and Their Gene CDS Haplotype (gcHap) Diversity in Rice

To identify candidate genes for detected ST QTLs, we searched all 35 QTL regions for possible candidate genes and found 25 harbor 43 cloned ST genes reported previously (Supplementary Table S4). To understand if and how the donor alleles at these candidate ST genes contributed to ST in the CY1 ILs, we performed a gcHap analysis on the 43 candidate ST genes in the 3000 core rice germplasm accessions [24,26] and the parents. Table S5 shows the gcHap diversity of the 43 ST genes.

## 5. Conclusions

Our results indicated that rice ST at the vegetative and reproductive stages had multiple complex phenological mechanisms controlled by large numbers of genes/QTLs. The Xian gene pool contains rich ‘hidden’ genetic variation for improving ST and yield potential, which could be efficiently exploited by selective introgression, including BC breeding, strong phenotypic selection and genome-wide characterization of donor introgression by marker genotyping and analyses. The developed ST ILs, plus their genetic information on the donor alleles for ST and yield traits, would provide a useful platform for developing superior ST and high-yield Geng varieties through breeding by design in the future.

## Figures and Tables

**Table 1:** Performances of nine promising CY1 introgression lines for seven yield traits under salt stress and non-stress conditions in the replicated progeny testing.

Lines	Donor	GYP (g) ^1^	SF(%)	FGN	SNP	TGW (g)	PN	PH (cm)	HD (Days)
A-11	Bg90-2	29.3	81.7	150.5	184.3	22.6	7.7	126.1	108.0
A-12	Bg90-2	40.1	92.9	163.5	176.1	22.5	9.7	117.7	108.0
A-21	Bg90-2	25.9	92.5	153.4	165.9	24.3	6.7	124.5	95.0
A-3	Bg90-2	27.3	95.3	153.6	161.2	26.2	7.0	127.3	112.0
B-5	X21	29.5	92.0	120.3	130.8	23.8	9.7	115.0	108.0
C-2	X22	28.0	87.0	132.5	152.3	26.7	8.3	127.1	104.0
C-30	X22	34.1	83.1	161.0	193.7	26.6	7.7	105.7	103.0
D-10	Q5	32.9	92.5	149.6	161.7	25.4	8.7	130.8	108.0
D-9	Q5	20.0	93.0	106.8	114.8	22.5	7.7	110.6	102.0
Mean		29.7	90.0	143.5	160.1	24.5	8.1	120.5	105.3
CY1		22.6	87.5	124.0	134.2	24.2	7.2	124.3	105.3
A-11	Bg90-2	12.8	63.0	473.0	187.8	23.5	4.0	96.0	107.0
A-12	Bg90-2	10.9	68.6	386.0	140.8	26.5	4.0	89.0	108.0
A-21	Bg90-2	14.6	73.5	476.0	162.0	27.5	4.0	93.0	107.0
A-3	Bg90-2	8.4	83.9	224.0	133.5	27.0	2.0	85.0	108.0
B-5	X21	9.4	66.0	285.0	72.0	23.3	6.0	76.0	118.0
C-2	X22	11.5	78.4	373.0	95.2	22.0	5.0	84.0	115.0
C-30	X22	13.9	73.2	583.0	132.7	23.3	6.0	118.0	106.0
D-10	Q5	11.1	81.1	344.0	84.8	23.3	5.0	98.0	110.0
D-9	Q5	11.7	63.1	352.0	93.0	26.3	6.0	91.0	112.0
Mean		11.6	72.3	388.4	122.4	24.7	4.7	92.2	110.1
CY1		3.5	54	48.8	99.4	20.3	3.6	85.5	117.0

^1^ GYP = grain yield per plant, SF = spikelet fertility, SNP = spikelet number per panicle, PN = panicle number per plant, TGW = 1000 grain weight, PH = plant height and HD = heading date, respectively.

## Data Availability

The original contributions presented in the study are included in the article/Supplementary Materials; further inquiries can be directed to the corresponding author/s.

## Supplementary Materials

The following supporting information can be downloaded at: https://www.mdpi.com/article/10.3390/cimb45060305/s1. Table S1: Grain yield trait values of 126 BC_2_F_4_ plants selected from five Chaoyou 1 (CY1) BC_2_F_4_ populations under salt stress from tillering to maturity. Table S2: Performance of grain yield traits of 67 salt-tolerant BC_2_F_5_ introgression lines selected from four BC_2_F_4_ populations under non-stress (N) and salt stress (S) conditions. Table S3: Correlation between yield-related traits of the 113 ILs from four populations evaluated under normal (the lower triangle) and salt stress (the upper triangle) conditions of the replicated progeny testing. Table S4: Identification of 58 QTLs for salt tolerance (ST) in 35 genomic regions (bins) across the rice genome detected in the 78 ST ILs from four BC_2_ CY1 populations. Table S5: QTLs affecting seven grain yield traits identified in low-(negative) or high (positive)-trait value ILs selected from four BC_2_F_5_ populations. Table S6: Summary of the negative selection for low-trait value ILs and positive selection for high-trait value introgression lines from the 113 initially selected for salt tolerance from four BC_2_ populations and the summary of the QTL mapping results for salt tolerance and yield traits by selective introgression. Figure S1: Genomic distribution of 58 loci (on the right side of chromosomes, Table S5) affecting seven yield traits identified by negative selection in the low-trait value introgression lines from four BC_2_F_4_ populations derived from inter-subspecific crosses between a Geng (japonica) variety, Chaoyou 1 (CY1), and four Xian (indica) donors. At all these QTLs, the donor alleles were associated with reduced trait values. Figure S2: Haplotype networks of 21 ST genes as candidate genes for 15 ST QTLs, the frequency distributions of major alleles (gcHaps) at each of the ST genes in five major rice populations (Adm, Aus, Bas, Geng, and Xian) of the 3000 rice core collections (Wang et al. 2018), and the associations of major alleles (gcHaps) at each of the loci with salt tolerance or susceptibility detected in ST introgression lines from four BC_2_ populations (Tables S4 and S7).

## References

[1] Ganie S.A., Molla K.A., Henry R.J., Bhat K., Mondal T.K. (2019). Advances in understanding salt tolerance in rice. Theor. Appl. Genet..

[2] Qin H., Li Y., Huang R. (2020). Advances and challenges in the breeding of salt-tolerant rice. Int. J. Mol. Sci..

[3] Peltier W.R., Tushingham A. (1989). Global sea level rise and the greenhouse effect: Might they be connected?. Science.

[4] Ismail A., Thomson M., Vergara G., Rahman M., Singh R., Gregorio G., Mackill D. (2010). Designing resilient rice varieties for coastal deltas using modern breeding tools. Tropical Deltas and Coastal Zones: Food Production, Communities and Environment at the Land and Water Interface.

[5] Quan R., Wang J., Yang D., Zhang H., Zhang Z., Huang R. (2017). EIN3 and SOS2 synergistically modulate plant salt tolerance. Sci. Rep..

[6] Xie Y., Xu P., Huang J., Ma S., Xie X., Tao D., Chen L., Liu Y.-G. (2017). Interspecific hybrid sterility in rice is mediated by OgTPR1 at the S1 locus encoding a peptidase-like protein. Mol. Plant.

[7] Pearson G.A., Ayers A., Eberhard D. (1966). Relative salt tolerance of rice during germination and early seedling development. Soil Sci..

[8] Noor A.U.Z., Nurnabi Azad Jewel G., Haque T., Elias S.M., Biswas S., Rahman M.S., Seraj Z.I. (2019). Validation of QTLs in Bangladeshi rice landrace Horkuch responsible for salt tolerance in seedling stage and maturation. Acta Physiol. Plant..

[9] Wing R.A., Purugganan M.D., Zhang Q. (2018). The rice genome revolution: From an ancient grain to Green Super Rice. Nat. Rev. Genet..

[10] Li Z.-K., Xu J.-L. (2007). Breeding for drought and salt tolerant rice (*Oryza sativa* L.): Progress and perspectives. Advances in Molecular Breeding Toward Drought and Salt Tolerant Crops.

[11] Mondal S., Septiningsih E.M., Singh R.K., Thomson M.J. (2022). Mapping QTLs for Reproductive Stage Salinity Tolerance in Rice Using a Cross between Hasawi and BRRI dhan28. Int. J. Mol. Sci..

[12] Batayeva D., Labaco B., Ye C., Li X., Usenbekov B., Rysbekova A., Dyuskalieva G., Vergara G., Reinke R., Leung H. (2018). Genome-wide association study of seedling stage salinity tolerance in temperate *japonica* rice germplasm. BMC Genet..

[13] Kumar P., Choudhary M., Halder T., Prakash N.R., Singh V., Vineeth V.T., Sheoran S., Longmei N., Rakshit S., Siddique K.H. (2022). Salinity stress tolerance and omics approaches: Revisiting the progress and achievements in major cereal crops. Heredity.

[14] Singh R., Mishra B., Chauhan M., Yeo A., Flowers S., Flowers T. (2002). Solution culture for screening rice varieties for sodicity tolerance. J. Agric. Sci..

[15] Jena K., Mackill D. (2008). Molecular markers and their use in marker-assisted selection in rice. Crop Sci..

[16] Calapit-Palao C.D.O. (2010). Identification of QTL for salinity tolerance at reproductive stage in rice (*Oryza sativa* L.). Master’s Thesis.

[17] Singh R.K., Kota S., Flowers T.J. (2021). Salt tolerance in rice: Seedling and reproductive stage QTL mapping come of age. Theor. Appl. Genet..

[18] Mohammadi R., Mendioro M.S., Diaz G.Q., Gregorio G.B., Singh R.K. (2013). Mapping quantitative trait loci associated with yield and yield components under reproductive stage salinity stress in rice (*Oryza sativa* L.). J. Genet..

[19] Chen C., Norton G.J., Price A.H. (2020). Genome-wide association mapping for salt tolerance of rice seedlings grown in hydroponic and soil systems using the Bengal and Assam Aus panel. Front. Plant Sci..

[20] Takehisa H., Shimodate T., Fukuta Y., Ueda T., Yano M., Yamaya T., Kameya T., Sato T. (2004). Identification of quantitative trait loci for plant growth of rice in paddy field flooded with salt water. Field Crops Res..

[21] Ammar M., Pandit A., Singh R., Sameena S., Chauhan M., Singh A., Sharma P., Gaikwad K., Sharma T., Mohapatra T. (2009). Mapping of QTLs controlling Na^+^, K^+^ and CI^−^ ion concentrations in salt tolerant *indica* rice variety CSR27. J. Plant Biochem. Biotechnol..

[22] Zhang G., Liu Y., Gui R., Wang Z., Li Z., Han Y., Guo X., Sun J. (2021). Comparative multi-omics analysis of hypoxic germination tolerance in weedy rice embryos and coleoptiles. Genomics.

[23] Murray M., Thompson W. (1980). Protocol of DNA isolation. Nucl. Acids Res..

[24] Shi J., Zhou X.-G., Yan Z., Tabien R.E., Wilson L.T., Wang L. (2021). Hybrid rice outperforms inbred rice in resistance to sheath blight and narrow brown leaf spot. Plant Dis..

[25] Wang W., Mauleon R., Hu Z., Chebotarov D., Tai S., Wu Z., Li M. (2018). Genomic variation in 3,010 diverse accessions of Asian cultivated rice. Nature.

[26] Hartl D.L., Clark A.G., Clark A.G. (1997). Principles of Population Genetics.

[27] Falconer D., Mackay T. (1996). Introduction to Quantitative Genetics.

[28] Li Z.-K., Fu B.-Y., Gao Y.-M., Xu J.-L., Ali J., Lafitte H., Jiang Y.-Z., Rey J.D., Vijayakumar C., Maghirang R. (2005). Genome-wide introgression lines and their use in genetic and molecular dissection of complex phenotypes in rice (*Oryza sativa* L.). Plant Mol. Biol..

[29] Zhang F., Zhai H.-Q., Paterson A.H., Xu J.-L., Gao Y.-M., Zheng T.-Q., Wu R.-L., Fu B.-Y., Ali J., Li Z.-K. (2011). Dissecting genetic networks underlying complex phenotypes: The theoretical framework. PLoS ONE.

[30] Zhang Z., Liu H., Sun C., Ma Q., Bu H., Chong K., Xu Y. (2018). A C2H2 zinc-finger protein OsZFP213 interacts with OsMAPK3 to enhance salt tolerance in rice. J. Plant Physiol..

[31] Shabala S., Alnayef M., Bose J., Chen Z.-H., Venkataraman G., Zhou M., Shabala L., Yu M. (2021). Revealing the role of the Calcineurin B-like protein-interacting protein kinase 9 (CIPK9) in Rice adaptive responses to salinity, osmotic stress, and K^+^ deficiency. Plants.

[32] You J., Zong W., Li X., Ning J., Hu H., Li X., Xiao J., Xiong L. (2013). The SNAC1-targeted gene OsSRO1c modulates stomatal closure and oxidative stress tolerance by regulating hydrogen peroxide in rice. J. Exp. Bot..

[33] Qingjie G., Qiuxiang L. (2007). Expression of rice OsAPX1 gene and its salt tolerance in tobacco. Mol. Plant Breed..

[34] Fang T., Dong Y., Li Y., Chen D., Chen X. (2015). The role of rice chloroplast ascorbate peroxidase in drought and high salt stress. Plant Physiol..

[35] Lee S.-K., Kim B.-G., Kwon T.-R., Jeong M.-J., Park S.-R., Lee J.-W., Byun M.-O., Kwon H.-B., Matthews B.F., Hong C.-B. (2011). Overexpression of the mitogen-activated protein kinase gene OsMAPK33 enhances sensitivity to salt stress in rice (*Oryza sativa* L.). J. Biosci..

[36] Liu X., Wang J., Yu Y., Kong L., Liu Y., Liu Z., Li H., Wei P., Liu M., Zhou H. (2019). Identification and characterization of the rice pre-harvest sprouting mutants involved in molybdenum cofactor biosynthesis. New Phytol..

[37] Li X., Yu B., Wu Q., Min Q., Zeng R., Xie Z., Huang J. (2021). OsMADS23 phosphorylated by SAPK9 confers drought and salt tolerance by regulating ABA biosynthesis in rice. PLoS Genet..

[38] Akbar M., Ponnamperuma F. (1982). Saline soils of South and Southeast Asia as potential rice lands. Rice Research Strategies for the Future.

[39] Lafitte H., Li Z., Vijayakumar C., Gao Y., Shi Y., Xu J., Fu B., Yu S., Ali A., Domingo J. (2006). Improvement of rice drought tolerance through backcross breeding: Evaluation of donors and selection in drought nurseries. Field Crops Res..

[40] Ali A., Xu J., Ismail A., Fu B., Vijaykumar C., Gao Y., Domingo J., Maghirang R., Yu S., Gregorio G. (2006). Hidden diversity for abiotic and biotic stress tolerances in the primary gene pool of rice revealed by a large backcross breeding program. Field Crops Res..

[41] He Y., Zheng T., Hao X., Wang L., Gao Y., Hua Z., Zhai H., Xu J., Xu Z., Zhu L. (2010). Yield performances of *japonica* introgression lines selected for drought tolerance in a BC breeding programme. Plant Breed..

[42] Meng L., Lin X., Wang J., Chen K., Cui Y., Xu J., Li Z. (2013). Simultaneous improvement in cold tolerance and yield of temperate *japonica* rice (*Oryza sativa* L.) by introgression breeding. Plant Breed..

[43] Zhang Q., Chen Q., Wang S., Hong Y., Wang Z. (2014). Rice and cold stress: Methods for its evaluation and summary of cold tolerance-related quantitative trait loci. Rice.

[44] Liang Y., Meng L., Lin X., Cui Y., Pang Y., Xu J., Li Z. (2018). QTL and QTL networks for cold tolerance at the reproductive stage detected using selective introgression in rice. PLoS ONE.

[45] Cui Y., Zhang W., Lin X., Xu S., Xu J., Li Z. (2018). Simultaneous improvement and genetic dissection of drought tolerance using selected breeding populations of rice. Front. Plant Sci..

[46] Jagadish S.K., Craufurd P.Q., Wheeler T. (2007). High temperature stress and spikelet fertility in rice (*Oryza sativa* L.). J. Exp. Bot..

[47] Liu C., Chen K., Zhao X., Wang X., Shen C., Zhu Y., Dai M., Qiu X., Yang R., Xing D. (2019). Identification of genes for salt tolerance and yield-related traits in rice plants grown hydroponically and under saline field conditions by genome-wide association study. Rice.

[48] Khatun S., Flowers T. (1995). Effects of salinity on seed set in rice. Plant Cell Environ..

[49] Fan X., Jiang H., Meng L., Chen J. (2021). Gene mapping, cloning and association analysis for salt tolerance in rice. Inter Natl. J. Mol. Sci..

[50] Khan M.A., Abdullah Z. (2003). Salinity–sodicity induced changes in reproductive physiology of rice (*Oryza sativa* L.) under dense soil conditions. Environ. Exp. Bot..

[51] Parida A.K., Das A.B. (2005). Salt tolerance and salinity effects on plants: A review. Ecotoxicol. Environ. Saf..

[52] Jaiswal S., Gautam R., Singh R., Krishnamurthy S.L., Ali S., Sakthivel K., Iquebal M.A., Rai A. (2019). Harmonizing technological advances in phenomics and genomics for enhanced salt tolerance in rice from a practical perspective. Rice.

[53] Weber D. (2009). Adaptive mechanisms of halophytes in desert regions. Salinity and Water Stress.

[54] Li Z.-K., Zhang F. (2013). Rice breeding in the post-genomics era: From concept to practice. Curr. Opin. Plant Biol..

[55] Kim J.H., Jang C.S. (2021). E3 ligase, the Oryza sativa salt-induced RING finger protein 4 (OsSIRP4), negatively regulates salt stress responses via degradation of the OsPEX11-1 protein. Plant Mol. Biol..

[56] Ren Z.H., Gao J.P., Li L.G. (2005). A rice quantitative trait locus for salt tolerance encodes a sodium transporter. Nat Genet..

